# The influence of moisture content variation on fungal pigment formation in spalted wood

**DOI:** 10.1186/2191-0855-2-69

**Published:** 2012-12-17

**Authors:** Daniela Tudor, Sara C Robinson, Paul A Cooper

**Affiliations:** 1 Faculty of Forestry, University of Toronto, 33 Willcocks Street, Toronto, M5S 3B3, Canada

**Keywords:** Fungal melanin, Pigment, Moisture content, Spalting

## Abstract

Eight fungal species known to produce wood pigmentation were tested for reaction to various moisture contents in two hardwood species. Fungal pigmentation by *Trametes versicolor* and *Xylaria polymorpha* was stimulated at low water concentrations in both *Acer saccharum* (sugar maple) and *Fagus grandifolia* (American beech), while *Inonotus hispidus* and *Polyporus squamosus* were stimulated above 22-28% and 34-38% moisture content in beech and in sugar maple respectively. *Fomes fomentarius* and *Polyporus brumalis* produced maximum pigmentation in beech at 26 - 41% and in sugar maple at 59 - 96% moisture content. The pink staining *Scytalidium cuboideum* pigmented both wood species at above 35% moisture content. This research indicates that controlling the moisture content values of wood substrates can stimulate the intensity of pigmentation of specific fungi when spalting wood for decorative and commercial purpose.

## Introduction

**Figure 1:**
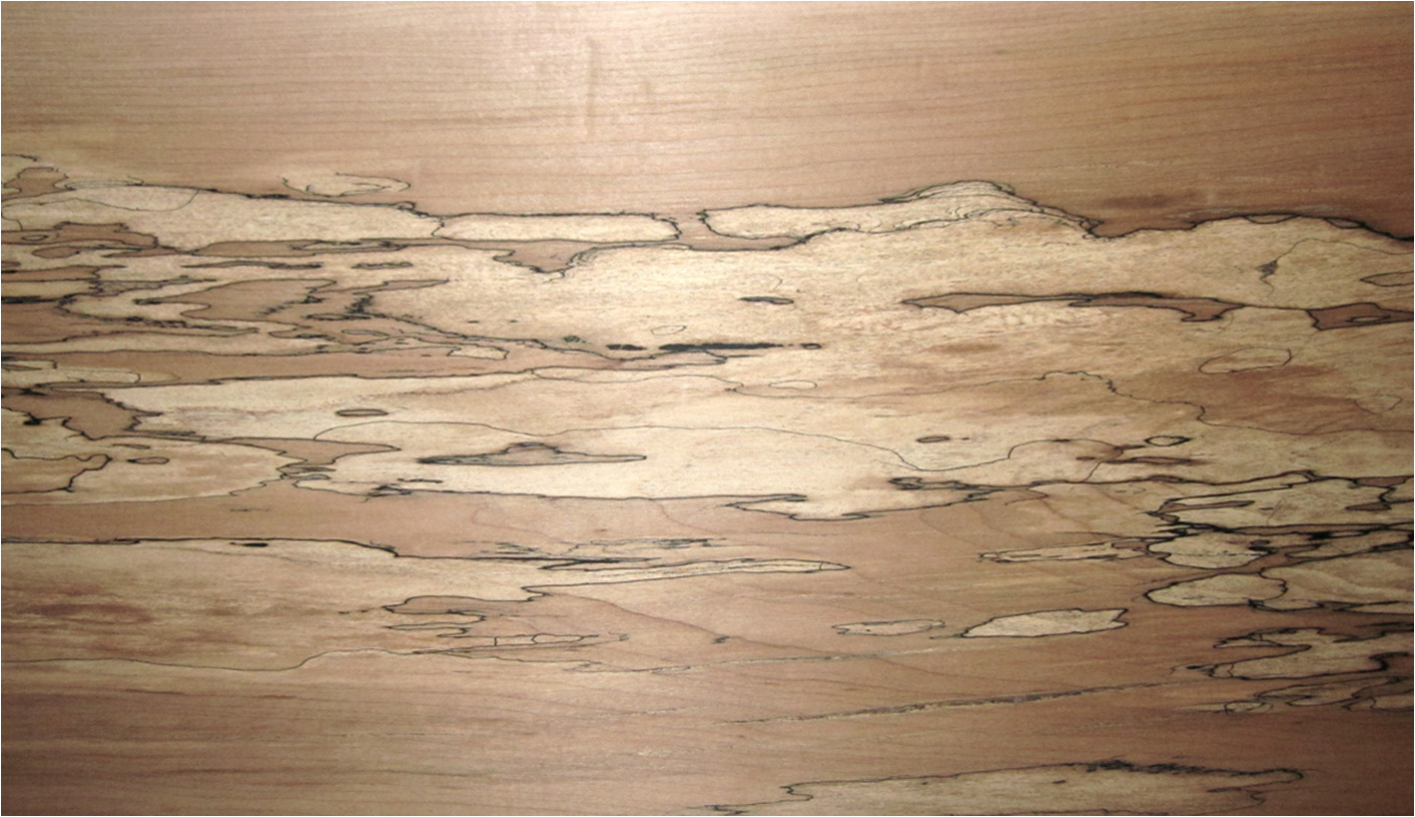
Patterns of zone lines and coloration characteristic in spalted wood.

Spalted wood is considered a value added product and can be produced by selected fungal inoculation of the wood substrate to create unique patterns (Robinson et al. 2007), and its considerable artistic and economic value is already established (Nicholls 2002; Donovan and Nicholls 2003a, b). The main characteristics of spalting are the spatial barrier demarcation of the fungal colonies by black pigment deposition, and the distinct colored zonation (Figure 1). The black pigment is a melanin type pigment (Butler and Day 1998) and it has a protective role against unfavorable environmental conditions (Campbell 1934; Pearce 1991; Henson et al. 1999). The stained wood of various colors is an effect of secondary metabolites in the form of pigmented fungicides, produced in the wood substrate by specific fungi (Margalith 1992; Duran et al. 2002). Wood moisture content (MC) is one of the most important conditions that influence fungal behavior and wood colonization patterns (Boddy 1983b). It is established that optimal fungal growth is achieved at 35–50 % MC on a dry weight basis, with a minimum required of 20–30 % necessary for fungal development; the values vary for different fungal species and inhabited wood substrates (Cartwright and Findlay 1958; Rayner and Todd 1979). Cartwright and Findlay (1958) also mention the ability of some fungal mycelium to survive below the fiber saturation point moisture content (26- 27% of the dry weight for most wood species), while spores, as well as mycelium of several fungal species, can survive for many years in dry condition (Schmidt 2006). Very high wood moisture content also inhibits fungal activity in wood substrate by limiting the quantity of the oxygen available in wood, preventing degradation (Cartwright and Findlay 1958; Boddy 1983a, b).

Moisture content of the substrate is not entirely dependent on the environmental conditions. It is known that wood-inhabiting fungi can influence the microclimatic regime in dead wood. Some fungi are able to regulate the moisture content of a substrate to ensure the optimal water availability, or as a strategy in antagonistic reactions, to create an environment stressful for fungal competitors, thereby avoiding combative exclusion (Boddy and Heilmann-Clausen 2008). Miller (1932) demonstrated that wood decomposer fungi like *Serpula lacrymans* (Wulfen) J. Schröt., *Schizopora paradoxa* (Schrad.) Donk and *Coniophora puteana* (Schumach.) P. Karst., could regulate the water content of the colonized habitat. During the initial stages of colonization, the dry conditions are improved for optimal growth by the mean of cellulose and polysaccharide decomposition, while at higher levels of humidity the water surplus is extracted from wood substrate into aerial mycelium, to ensure the optimal moisture content. By virtue of pigmented zonation lines that they produce, fungi from *Xylaria* species seem to maintain wood drier than ambient conditions (Boddy et al. 1989; Heilmann-Clausen 2001), while *Armillaria* species occupy wood wetter than ambient (Lopez-Real and Swift 1975; Chapela and Boddy 1988). As reported by Humar et al. (2001), moisture content of wooden blocks inoculated with *Trametes versicolor* (L.) Lloyd rose from 10 % to 57 % above the fiber saturation point after 12 weeks of exposure to decay, and opportunistic mold fungi like *Penicillium* spp. can ensure fungal succession on various wood substrates by improving moisture content in decayed wood (Dix 1985). However, various water content levels determine the prevalence of fungal species inhabiting the wood substrate. Based on the moisture content preference, Käärik (1974) indicate three distinctive groups that colonize wood: fungi that occupy wood at 37 – 47 % moisture content, fungi that prefer a drier part of the wood of 17-23% MC, and fungi that had no restriction in terms of water availability.

There are 44 species of basidiomycetes and 10 species of ascomycetes that have been reported to form dark zone lines (Lopez-Real and Swift 1975). Research by Hubert(1924) indicated that fungal melanin deposition in spatial demarcation could be triggered by limited water availability. The anticipation of desiccation might cause fungal species like *Xylaria polymorpha* (Pers.) Grev., *Bjerkandera adusta* (Willd.) P. Karst., *Phellinus igniarius* (L.) Quél. and *Porodaedalea pini* (Brot.) Murrill to develop an effective strategy to ensure the survival of the colonies. They produce high resistance, melanin-type pigment that surrounds the fungal community like a barrier, blocking the water exchange within the wood substrate. This may appear as fine delimitation lines in section. Campbell (1933), in his study on zone line formation by *X. polymorpha*, refers to desiccation as the cause of a particular kind of zone lines, as well as for *Armillaria mellea* (Vahl) P. Kumm (Campbell 1934).

Laboratory studies by Lopez-Real and Swift (1975, 1977) indicate that melanized mycelium formation by *A. mellea* and *Stereum hirsutum* (Willd.) Pers., is considered as a normal morphogenetic process, and occurs at any moisture content when growth is possible, except for *S. hirsutum* at low humidity (< 35% MC), when pigmentation is inhibited at early stages of growth. Studies by Campbell (1933, 1934), Lopez-Real (1975), Rayner and Todd (1979), Boddy (1986), and Boddy et al. (1989) describe the formation of melanized mycelium in zone lines, in inter- and intraspecific antagonistic reactions, and offer a perspective on substrate and environmental conditions that influence such formations in natural settings. Lopez-Real and Swift (1975) indicated the existence of a relationship between the moisture content of the substrate during the initial period of colonization and the ability of *Armilaria mellea* to form melanin*.* For *Stereum hirsutum*, only high moisture content of the substrate may be a critical factor determining pigment formation*.*Campbell (1933, 1934) studied *in vitro* zonation of *A. mellea* and *X. polymorpha*, mentioning that optimum moisture is necessary to produce pigmentation; however no specific values were indicated. Rishbeth (1951) studied the behavior of *Heterobasidion annosum* (Fr.) Bref. indicating that infection is critical in freshly cut stumps. Hopp (1938) investigated pigmentation of *Ganoderma applanatum* (Pers.) Pat. and *Phellinus igniarius* (L.) Quél. and found that moisture content strongly influences the antagonistic reactions with pigment production in wood. However, research under laboratory conditions is limited, and a more elaborate and consistent investigation was necessary to determine the optimal conditions for pigmentation for given wood species by fungal species utilized in spalting. This study investigates the direct influence of moisture content of specific wood substrates on fungal pigmentation, and the results are important for manipulating fungal pigmentation for spalting production. The ability to manipulate fungal reactions through moisture content changes in wood, offers a chance to enhance the pigmentation intensity and patterns currently available with spalted woods. It also offers an opportunity to add considerable value to underutilized hardwood species.

## Material and Methods

### Wood and fungal species selection

Two common wood species from southern Ontario, sugar maple (*Acer saccharum* Marsh) and beech (*Fagus grandifolia* L.) were selected for testing, based on their contrasting natural spalting prevalence. The average oven-dry specific gravity (SG) of the tested wood species was SG=0.68 for sugar maple and SG=0.74 for beech.

Eight fungal species were selected based on their spalting ability (Table 1). The ascomycete *X. polymorpha* is known to produce melanin by a polyketide pathway and the basidiomycete *T. versicolor* probably produces melanin by the catechol pathway (Bell and Wheeler 1986; Taylor et al. 1988). For those fungi, we tested three strains each to determine whether melanin production varied significantly within a species. *X. polymorpha* strains UAMH 11518, UAMH 11519, UAMH 11520 had been isolated from *Acer saccharum* in Alberta, MI, USA. Two *T. versicolor* strains were obtained from the Forest Products Laboratory in Madison, WI, USA: Mad 697 was, isolated from a cankered area of *Fagus grandifolia* in Vermont, USA, and strain R105 was isolated from a dead branch of *Malus sp.* in New York, USA. The third strain UAMH 11521 was isolated from *Acer saccharum* in Houghton, MI, USA.

**Table 1:** Wood and fungal species selection

**Wood species/specific gravity (SG)**	**Fungal species/Culture collection number**	
Sugar maple/SG=0.68	*Xylaria polymorpha*	UAMH 11518
		UAMH 11519
		UAMH 11520
Beech/SG=0.74	*Trametes versicolor*	Mad 697
		FP 72074-R
		UAMH 11521
	*Polyporus squamosus*	UAMH 11653
	*Polyporus brumalis*	UAMH 11652
	*Fomes fomentarius*	UAMH 11654
	*Inonotus hispidus*	F2037
	*Piptoporus betulinus*	UAMH 11651
	*Scytalidium cuboideum*	UAMH 4802

Other fungal species, one strain per species, were used for additional experiments: *Polyporus squamosus* (Huds.) Fr. UAMH 11653 isolated from beech in Toronto, ON, Canada, *Polyporus brumalis* (Pers.) Fr. UAMH 11652 isolated from unidentified dead wood in Toronto area, ON, Canada, *Fomes fomentarius* (L.) J.J. Kickx UAMH 11654 isolated from birch in Toronto, ON, Canada, *Inonotus hispidus* (Bull.) P. Karst. F2037 of unknown origin, *Piptoporus betulinus* UAMH 11651 (Bull.) P. Karst., isolated from sugar maple in Toronto, ON, Canada. One staining fungus investigated for pigment formation was *Scytallidium cuboideum* (Sacc. & Ellis) Sigler & Kang UAMH 4802 isolated from Na-pentachlorophenate-dipped red oak lumber.

Fungal cultures used for inoculation were grown on 95x15 mm Petri dishes with 2% malt extract agar at room temperature for two weeks prior inoculation.

## Test procedure

### Moisture content test preparation

The moisture content test was performed using a modified decay jar test with vermiculite instead of soil, as outlined in Robinson et al. (2009, 2012), to avoid eventual influence of soil substrates on pigment formation. Jars with plastic lids (250 ml) were prepared with 15g vermiculite and variable amounts of distilled water per set. According to AWPA (2009) Standards, the amount of water added in the jars should be 130% of the water-holding capacity (WHC) of the substrate, in our case vermiculite, for optimum condition of decay. Based on vermiculite WHC, our calculations indicate that 30g of distilled water should be added in each cultured jar for standard incubation conditions. Nine levels of water availability was tested, modifying the amount of water added to each jar, in increments of 5g, five levels under and three levels above the standard conditions for decay (5g, 10g, 15g, 20g, 25g, 30g, 35g, 40g, and 45g).

### Inoculation and incubation

Culture jars with vermiculite and the specified amount of distilled water were autoclaved for 30 minutes at 121°C. Five replicates per set of 14 mm wood cubes, preconditioned at 50°C to provide an initial dry weight, were surface steam sterilized for 30 minutes under atmospheric pressure at 100°C. After cooling, the wood samples were placed in culture jars, and inoculated with a 0.5x2 cm strip of fungal inoculum. Culture jars were incubated at 27°C ± 2°C and 80% ± 5% relative humidity in the inoculation chamber, for eight weeks for *T. versicolor* and *F. fomentarius* and ten weeks for the remaining fungi. At the end of the period of incubation, blocks were removed from jars, gently cleaned at surface to remove mycelium and any traces of vermiculite, and weighed before and after overnight oven drying to determine final moisture content and mass loss. To avoid changes of fungal pigment colors, the drying temperature was modified to 50°C instead of 103°C standard for MC testing.

### Pigment assessment

Dried wood samples were scanned externally and internally with Epson WorkForce 500 scanner at 2400 dpi. The obtained images were analyzed for pigment evaluation with Scion Image software, following the protocol described in Robinson et al*.* (2009a).

To analyze the importance of moisture content for fungal pigment formation, a one-way ANOVA was run, followed by Tukey’s HSD at α = 0.05, using SAS, version 9.2, with fungal inoculation and induced conditions as independent variables, for each wood species.

### Estimation of initial conditions and changes of substrate moisture content

To estimate the wood moisture content in conditions simulated in the experiment, two sets of tests were run for eight weeks, without fungal inoculation, under identical conditions as the main test. Five replicates of wood samples per treatment, initially oven-dried conditioned, were weighed every day for the first week and once every week for the following period, to monitor changes of moisture content in wood samples. To minimize mold contamination, wood samples were manipulated and weighted in a sterile laminar flow hood. However, for comparison, wood samples from a second set were kept in sterile conditions and weighed only at the end of the eight-week incubation period. Wood moisture content was calculated based on the final oven-dry weight.

## Results

### Initial conditions

**Figure 2:**
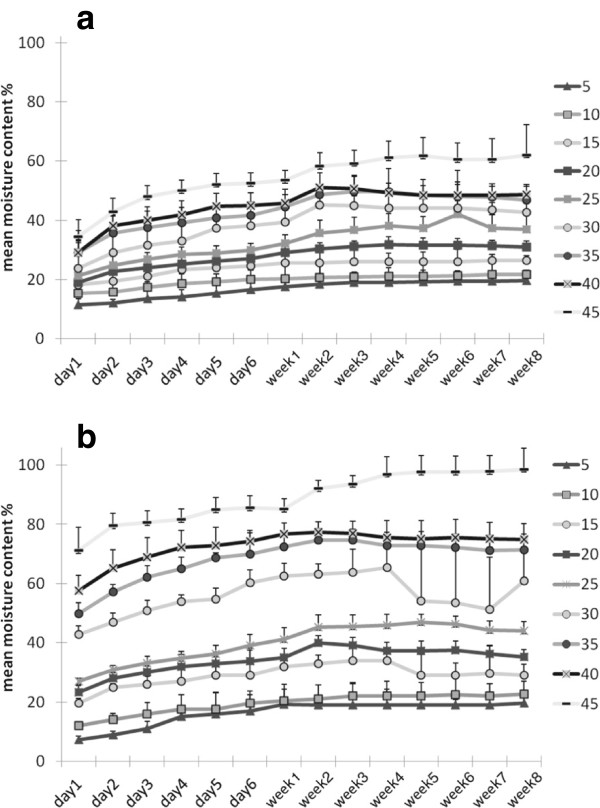
Initial moisture content in beech (a) and sugar maple (b) incubated in culture condition without fungal inoculation, measured over eight weeks period (unsterile conditions).

Periodical measurement of beech and sugar maple samples kept in culture conditions without fungal inoculation, showed that after one day, wood samples attained 70% of the final moisture content measured at the end of eight weeks; after four days, samples reached approximately 80% of the final moisture content, and after approximately two weeks, wood samples reached moisture content equilibrium (Figure 2). There are clear differences among treatments at every stage of testing period.

Comparisons between the final results of wood samples exposed to the same moisture content settings, but kept in sterile and unsterile conditions, indicate a slightly positive difference in water uptake of the sterile wood samples, that might be explained by water loss due to periodic handling for weight measurements. Also, the results indicate that sugar maple’s hygroscopic capacity was greater than that of beech, for the same amount of water availability (Table 2).

**Table 2:** **Final moisture content in wood samples (five replicates per set) incubated with various fungal species: tv = *T. versicolor *(average of three strains); xp = *X. polymorpha *(average of three strains)*; *ih = *I. hispidus*; pbr = *P. brumalis*; pbt = *P. betulinus; *ff = *F. fomentarius*; psq = *P. squamosus*; sc = *S. cuboideum*; - = no fungal inoculation**

	**Fungal species**	**tv**	**xp**	**ih**	**pbr**	**pbt**	**ff**	**psq**	**sc**	**-**
	**Treatment g water/jar**	**Mean % moisture content /standard deviation**								
Beech	5	27 /5	21 /2	23 /5	27 /3	31 /2	27 /3	25 /5	13 /4	21 /3
	10	34 /4	33 /4	32 /4	33 /3	31 /2	31 /2	30 /2	23 /10	25 /3
	15	37 /6	35 /3	34 /5	37 /4	38 /4	34 /8	33 /5	33 /3	29 /3
	20	39 /3	40 /6	44 /5	41 /2	40 /3	43 /4	40 /5	43 /4	37 /4
	25	45 /8	43 /6	50 /6	44 /4	53 /3	53 /7	51 /7	46 /5	40 /4
	30	46 /7	49 /5	54 /7	44 /2	52 /3	56 /4	58 /5	47 /3	50 /3
	35	50 /10	50 /5	55 /5	45 /8	55 /9	52 /7	58 /14	47 /3	56 /4
	40	58 /11	54 /5	58 /2	55 /6	53 /5	52 /11	62 /13	47 /5	59 /4
	45	63 /14	60 /6	79 /8	59 /9	62 /2	77 /16	88 /10	49 /5	59 /3
Sugar maple	5	24 /5	23 /3	19 /4	29 /3	18 /7	8 /3	24 /1	9 /4	19 /4
	10	33 /5	31 /3	33 /3	33 /5	35 /3	10 /2	29 /1	15 /1	22 /2
	15	37 /6	39 /8	36 /6	36 /3	46 /2	13 /2	31 /4	20 /3	31 /2
	20	41 /4	46 /3	66 /11	52 /6	66 /3	23 /4	34 /3	25 /2	36 /2
	25	47 /7	51 /3	71 /9	59 /6	80 /8	24 /6	43 /2	25 /4	44 /2
	30	47 /6	56 /5	77 /5	72 /4	91 /5	37 /6	44 /4	31 /4	59 /13
	35	55 /6	57 /5	81 /9	82 /3	95 /6	47 /5	45 /4	37 /5	70 /11
	40	76 /8	71 /7	89 /7	85 /2	96 /7	53 /3	48 /2	37 /3	82 /6
	45	89 /8	78 /6	90 /4	87 /4	93 /3	54 /3	58 /3	37 /2	88 /8

### Mass loss

**Figure 3:**
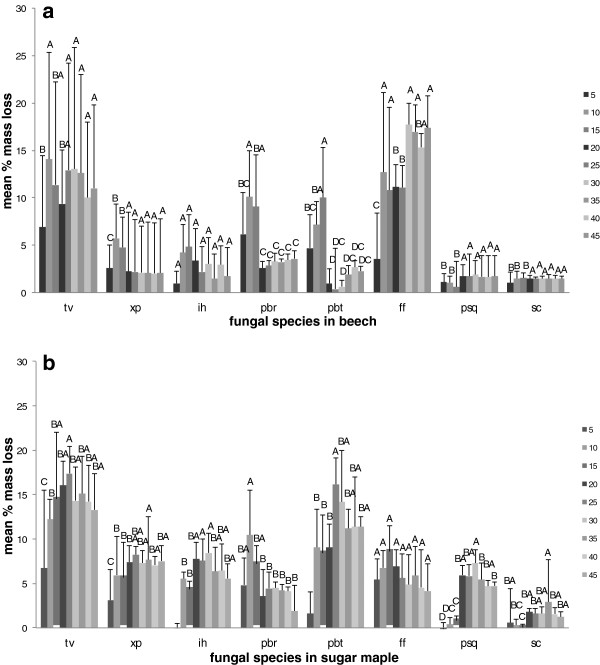
**Mass loss of beech (a) and sugar maple (b) wood samples incubated with various fungal species: tv = *T. versicolor *(average of three strains); xp = *X. polymorpha *(average of three strains); ih = *I. hispidus*; pbr = *P. brumalis*; pbt = *P. betulinus; *ff = *F. fomentarius*; psq = *P. squamosus*; sc = *S. cuboideum.*** Error bars represent one standard deviation of five replicates per set. Different letters represent significant differences at α = 0.05 within each category.

None of the treatments significantly influenced mass loss in beech and sugar maple. However, lower levels of moisture content attained by treatment with 5, 10 and 15 g of water resulted in stimulation of mass loss in beech inoculated with *X. polymorpha, P. brumalis* and *P. betulinus* (Figure 3a). Mass loss induced by *F. fomentarius* was greater in beech samples, while the rest of the fungi degraded sugar maple samples more (Figure 3a and b).

### Influence of induced condition on pigment formation

**Figure 4:**
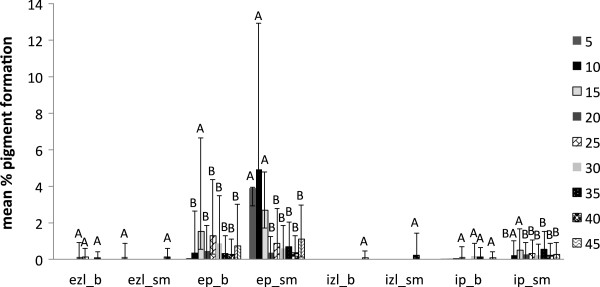
**Pigment production in beech (b) and sugar maple (sm) by *Trametes versicolor *(average of three strains) at various moisture content values in eight weeks of incubation: ezl – external zone lines; ep – external pigmentation; izl – internal zone lines; ip – internal pigmentation.** Error bars represent one standard deviation of five replicates per set. Different letters represent significant differences at α = 0.05 within each category.

External black to dark brown pigmentation by *T. versicolor* (average of three strains) was higher with the low water availabilities with maximum pigmentation at treatment with 10 g of water for sugar maple and 15 g water for beech; these treatments resulted in similar initial conditions of approximate 30 % moisture content for both wood species tested. Internal pigmentation in sugar maple was also stimulated by low water content. The large standard deviations for the degree of pigmentation (Figure 4) reflect the high variability among the strains used for the moisture content experiment.

**Figure 5:**
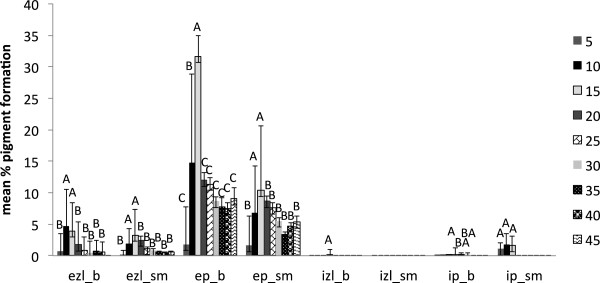
**Pigment production in beech (b) and sugar maple (sm) by *Xylaria polymorpha *(average of three strains) at various moisture content values in ten weeks of incubation: ezl – external zone lines; ep – external pigmentation; izl – internal zone lines; ip – internal pigmentation.** Error bars represent one standard deviation of five replicates per set. Different letters represent significant differences at α = 0.05 within each category.

External zone lines and black pigmentation produced by *X. polymorpha* (average of three strains) in beech and sugar maple was significant at treatments with 10 and 15 g of water for both wood species (P<0.0001). Internal pigmentation was highest in sugar maple at low moisture content (Figure 5). Unlike *T. versicolor,* this fungus demonstrated smaller deviations among the data and a greater consistency in pigment formation among the three strains.

**Figure 6:**
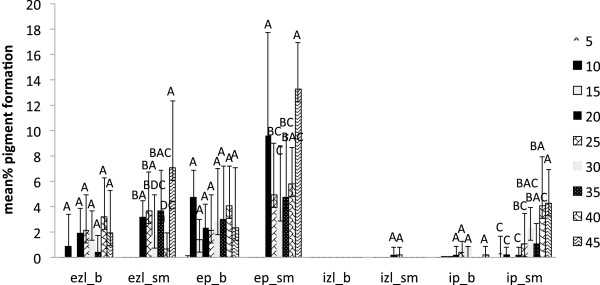
**Pigment production in beech (b) and sugar maple (sm) by *Inonotus hispidus *at various moisture content values in eight weeks of incubation: ezl – external zone lines; ep – external pigmentation; izl – internal zone lines; ip – internal pigmentation.** Error bars represent one standard deviation of five replicates per set. Different letters represent significant differences at α = 0.05 within each category.

**Figure 7:**
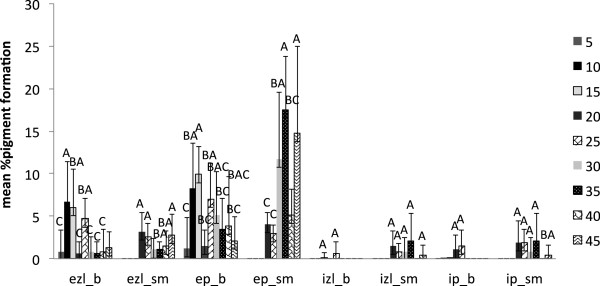
**Pigment production in beech (a) and sugar maple (b) by *Polyporus squamosus *at various moisture content values in eight weeks of incubation: ezl – external zone lines; ep – external pigmentation; izl – internal zone lines; ip – internal pigmentation.** Error bars represent one standard deviation of five replicates per set. Different letters represent significant differences at α = 0.05 within each category.

In the case of *I. hispidus* and *P. squamosus*, there was no significant moisture content treatment condition that enhanced pigmentation; however, there was a tendency of enhanced dark brown pigmentation observed at higher levels of moisture content (Figures 6 and 7).

**Figure 8:**
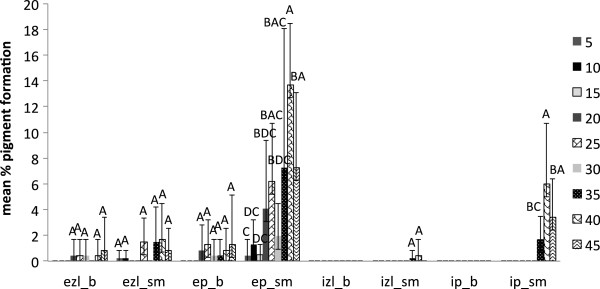
**Pigment production in beech (a) and sugar maple (b) by *Fomes fomentarius *at various moisture content values in eight weeks of incubation: ezl – external zone lines; ep – external pigmentation; izl – internal zone lines; ip – internal pigmentation.** Error bars represent one standard deviation of five replicates per set. Different letters represent significant differences at α = 0.05 within each category.

**Figure 9:**
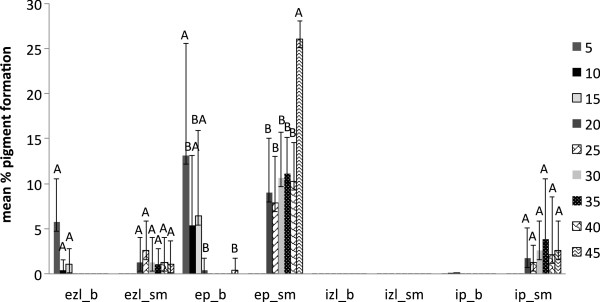
**Pigment production in beech (a) and sugar maple (b) by *Polyporus brumalis *at various moisture content values in eight weeks of incubation: ezl – external zone lines; ep – external pigmentation; izl – internal zone lines; ip – internal pigmentation.** Error bars represent one standard deviation of five replicates per set. Different letters represent significant differences at α = 0.05 within each category.

*Polyporus brumalis* had significant black external pigmentation in sugar maple at treatments with 45g of water (P<0.0001), while in beech the pigmentation was stimulated at lower levels of moisture content. The same tendency was observed for *F. fomentarius* (Figures 8 and 9)*. Piptoporus betulinus,* the only brown rot fungus investigated, produced no pigmentation in either wood species tested.

**Figure 10:**
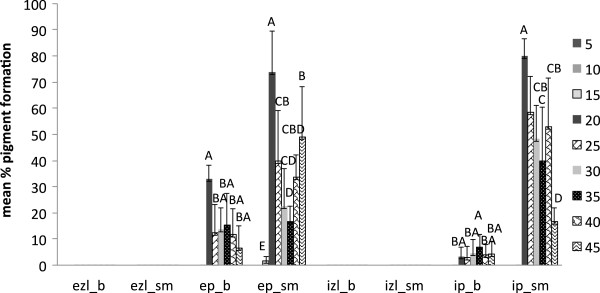
**Pigment production in beech (a) and sugar maple (b) by *Scytallidium cuboideum *at various moisture content values in eight weeks of incubation: ezl – external zone lines; ep – external pigmentation; izl – internal zone lines; ip – internal pigmentation.** Error bars represent one standard deviation of five replicates per set. Different letters represent significant differences at α = 0.05 within each category.

The red stain fungus *S. cuboideum* had significant external pigmentation at treatments with 20 g of water for both wood species tested (P<0.0001) at eight weeks of incubation, and there was also significant internal pigmentation in sugar maple (P<0.0001) at the same treatment (Figure 10).

## Discussion

For commercial and artistic purposes, spalting should result in a high intensity of wood pigmentation, while minimizing the loss in strength and integrity of the wood after exposure to fungal activity. The natural wood resistance to decay is influenced by many other factors such as wood extractives and ambient temperature. In our tests, wood integrity was reflected by mass loss measurements at the end of period of incubation. As expected, based on the specific gravity of the wood species tested, sugar maple samples proved to have higher degradability than beech, with few exceptions. *Polyporus brumalis* and *S. cuboideum* degraded both wood species at the same rate, and *F. fomentarius* was more effective for beech degradation, within the same treatments of water availability. However, sugar maple is more prone to spalting, as the occurrence of fungal pigmentation and zone line is much higher than in beech.

The moisture content in wood substrates can fluctuate considerably, and is influenced by the relative humidity of the microclimate, the substrate hygroscopicity, and fungal decomposition activity (Chapela and Boddy 1988; Boddy et al. 1989; Heilmann-Clausen 2001; Boddy and Heilmann-Clausen 2008). From the analysis of moisture content of wood samples exposed to the same testing condition, without fungal inoculation, it was determined that sugar maple was more hygroscopic than beech (Figure 2), suggesting higher hemicelluloses, lower lignin and/or lower extractive content (Rowell 2012). For the present study, it was also important to establish the predicted value of the initial moisture content that the wood substrate was able to achieve for each treatment (see Table 2).

Due to periodic removal of wood samples for weighing, the measured MC of the wood samples varied over the experiment period. This variation could be explained either by loss of moisture through evaporation, or by changes to the position of the wood samples in the vermiculite. However, comparison of final results of this experiment with another set of sterile wood samples, kept in the same condition and measured only for final moisture content after eight weeks, indicates that the final MC of the two sets of wood samples are comparable (results not shown).

The most favorable condition for the growth of fungi in wood is slightly above the fiber saturation point (FSP: 25-30% MC), when free diffusion of enzymes can take place within the film of liquid water that coat the cell walls, but where some air spaces remain for gas diffusion (Cartwright & Findlay 1958). According to Lopez-Real and Swift (1975), the formation of black pigmented delimitation zones requires a similar situation, where free water still exists in the lumina of the wood cells, and the levels of concentration of CO_2_ are above atmospheric conditions, with the presence of atmospheric levels of oxygen at least for the initial exposure.

However, the present study shows that the reaction of fungal species investigated at different values of water availability varied considerably, even between the two wood species studied. *Trametes versicolor* produced pigmentation at a lower moisture content equivalent of 18–35 % for sugar maple, and 26–32 % for beech (Figure 4), with no inhibition of mass loss. The ascomycete *X. polymorpha* preferred a moisture content that was more consistent between the two wood species studied, producing maximum pigmentation at 29–33 % in sugar maple and 29–32 % initial moisture content in beech (Figure 5). However, the same moisture content that stimulated pigmentation in beech, also resulted in the highest mass loss (Figure 3a). In the case of *I. hispidus* the pigmentation was inhibited at low moisture contents, under 22-28% in beech and 34-38% in sugar maple (Figure 5), and no treatment had a significant influence on mass loss. The same trend can be recognized in *P. squamosus* (Figure 7), while *F. fomentarius* and *P. brumalis* both show a completely different tendency, with maximum pigmentation in beech at 26–41 % and in sugar maple at 59- 96% (Figures 8 and 9). The pink staining fungus *S. cuboideum* developed more pigmentation at an the initial moisture content above 35 % for both wood species (Figure 10). The differences in the reaction among fungal species to various moisture contents might be the outcome of fungal specificity to certain conditions and substrate constraints, as part of successional colonization that ensure wood decomposition (Boddy 1983a; Boddy and Rayner 1983; Cooke and Rayner 1984; Boddy 1986; Rayner 1986; Boddy et al. 1989). It is also known that many of the studied fungi produce different types of black pigmentation, using various phenolic compounds from wood substrates as precursors for melanin biosynthesis (Bell and Wheeler 1986; Butler et al. 2001). Those cumulative observations emphasize once more the complex interactions of fungal species in wood decay.

This research indicates that spalting could be stimulated by controlling moisture content of the substrate, and optimal conditions are specific to wood and fungal species. Pigmentation was stimulated at low water concentrations in the case of *T. versicolor* and *X. polymorpha* for both wood species tested, while *I. hispidus*, *P. squamosus*, *P. brumalis* and *S. cuboideum* showed a tendency of enhanced pigmentation at higher levels of moisture content (35%-55%). Moisture content amounts tested did not significantly affect wood mass loss.

For direct applicability in industrial spalting production, fungal strains used in production should be carefully tested to assess the maximum pigmentation ability in regard with MC. Pairing of fungal strains should be considered based on their similarity of pigment production at proximate MC values. A special consideration should be given to fungi like *X. polymorpha* that produce maximum pigmentation at lower values of MC, and proved to be more consistent in strain variation. To overcome the limited surface that fungi can inhabit at low water availability due to restrained growth, a MC optimal for growth should be initially induced, followed by a drying period to stimulate pigmentation.

## Abbreviations

MC: Moisture content; WHC: Water-holding capacity.

## Competing interests

The authors declare that they have no competing interests.
